# Supplementary material for: The causal relationship between the human gut microbiota and pyogenic arthritis: a Mendelian randomization study
Source: Front Cell Infect Microbiol. 2024 Nov 26;14:1452480. doi: 10.3389/fcimb.2024.1452480 (PMC11629706; doi:10.3389/fcimb.2024.1452480)
Supplement: Supplementary file 1 [file DataSheet1.zip › mendelian test/ebi-a-GCST90027736.csv_forest.pdf]

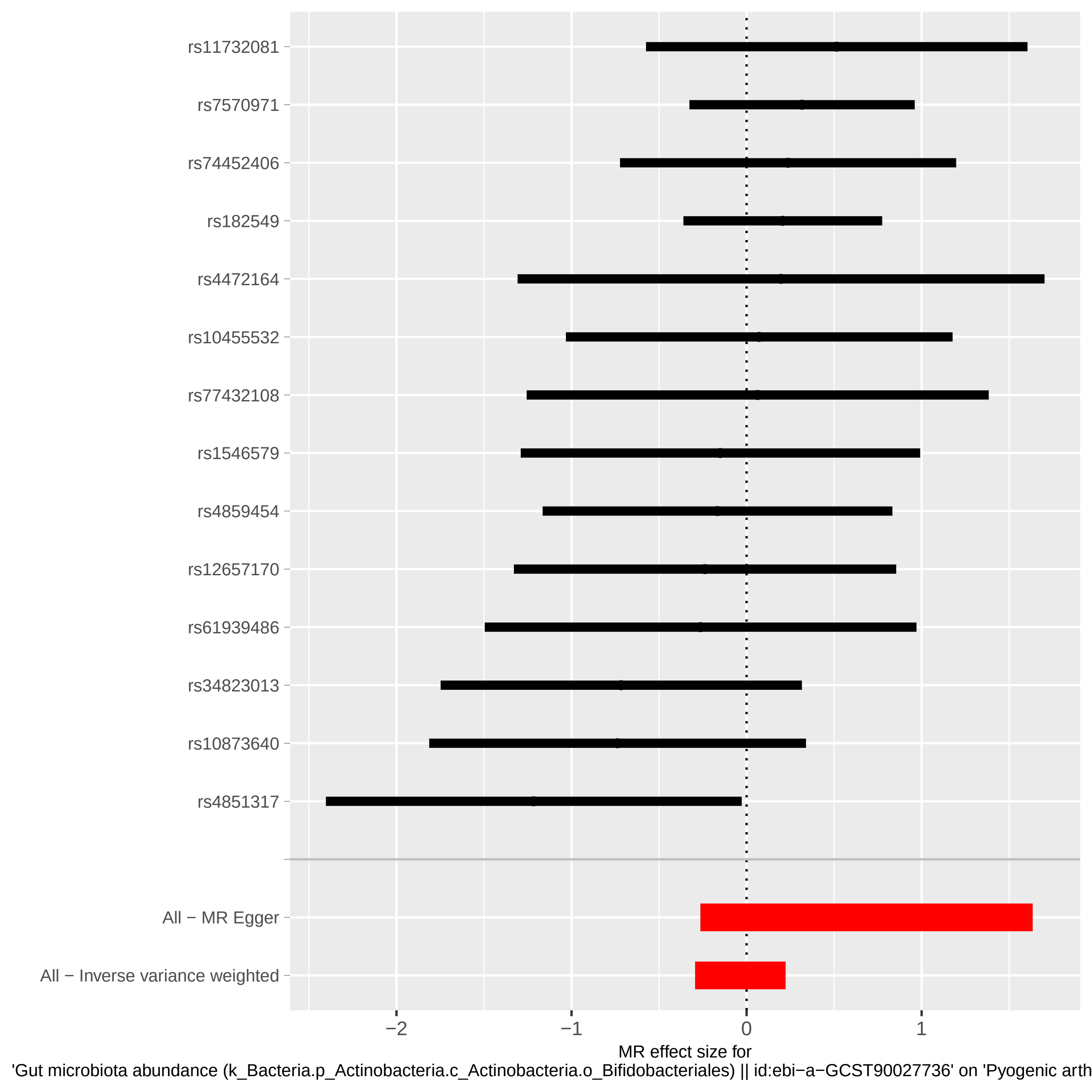

rs11732081
rs7570971
rs74452406
rs182549
rs4472164
rs10455532
rs77432108
rs1546579
rs4859454
rs12657170
rs61939486
rs34823013
rs10873640
rs4851317
All − MR Egger
All − Inverse variance weighted
−2
−1
0
1
MR effect size for
'Gut microbiota abundance (k_Bacteria.p_Actinobacteria.c_Actinobacteria.o_Bifidobacteriales) || id:ebi−a−GCST90027736' on 'Pyogenic arth
